# Case report of simultaneous presentation of pulmonary embolism and pericardial effusion following an oncological esophagectomy

**DOI:** 10.1016/j.ijscr.2020.10.090

**Published:** 2020-10-28

**Authors:** Daniela Jou-Valencia, Frederieke A. Dijkstra

**Affiliations:** Department of Surgery, University of Groningen, University Medical Center Groningen (UMCG), Groningen, the Netherlands

**Keywords:** Esophageal cancer, Esophageal resection, Case report, Pulmonary embolism, Pericardial effusion, Direct oral anticoagulant (DOAC)

## Abstract

**Introduction:**

This is the first reported case of simultaneous presentation of pulmonary embolism and pericardial effusion following esophagectomy. This case illustrates a diagnostic and therapeutic challenge exemplifying the difficulties arising from complex anticoagulant considerations in esophageal cancer.

**Patient case:**

A 72 year old male undergoes an oncological esophageal resection. Postoperatively the patient develops pulmonary embolism for which he is treated with Rivaroxaban. After starting Rivaroxaban the patient develops a large pericardial effusion.

**Discussion:**

We suspect that the treatment of pulmonary embolism with Rivaroxaban had a causative role in the development of pericardial effusion. Based on literature we suspect that chemoradiotherapy increased susceptibility.

**Conclusion:**

Diagnosis and treatment of simultaneous pulmonary embolism and pericardial effusion remains a challenge. Special consideration should be taken when using Rivaroxaban in esophageal cancer patients; this should always be conducted in consultation with a coagulation specialist.

## Highlights

• First reported case of simultaneous pulmonary embolism and pericardial effusion following esophageal resection.
• Contradicting anticoagulation indications poses a therapeutic challenge.
• Rivaroxaban as possible cause of pericardial bleed.

## 1 Introduction

Esophageal cancer (EC) is a serious diagnosis with high morbidity and mortality. Treatment involves chemoradiotherapy and esophageal resection. This is a complex procedure with a high risk of complications [1].

We present a case of a 72-year-old male with progressive chest pain (CP) and shortness of breath (SoB) following an uncomplicated esophagectomy in a University Medical Center in the Netherlands. The symptoms were initially attributed to a massive pulmonary embolism (PE) for which Rivaroxaban was started. Six days after, a pericardial effusion (PcE) was seen on CT alongside the PE.

This is the first reported case of the simultaneous presentation of PE and PcE following esophagectomy. This case illustrates a diagnostic and therapeutic challenges, exemplifying the difficulties arising from complex anticoagulant considerations in EC.

The case is presented according to SCARE criteria [2].

## 2 Presentation of case

A 72 year-old Caucasian male (Table 1) was diagnosed with distal esophageal squamous cell carcinoma (cT2N1M0) for which he received carboplatin/paclitaxel chemoradiotherapy. After 5 cycles, positron emission tomography showed complete response to therapy. Due to the aggressive nature of the cancer, an esophageal resection was indicated. Twelve weeks after the last chemoradiotherapy session the patient underwent a robot-assisted minimally-invasive esophagectomy with intra-thoracic anastomosis. Procedure was executed by two experienced upper GI surgeons.

**Table 1:** Patient Characteristics.

**Baseline characteristics**		
Age	72 years	
Gender	Male	
Race	Caucasian	
BMI	2596 kg/m^2^	
**Medical History**		
2001	Myocardial infarction	Percutaneous Transluminal coronary angioplasty (PTCA) of circumflex artery
2003/12	Peripheral T-Cell Non-Hodgkin Lymphoma Stadium 4B: Cervical, mediastinal and inguinal	8x CHOP chemotherapy (after 6 sessions complete remission)
2004/09	Recurrence T-cell Non-Hodgkin Lymphoma	DHAP/VIM/DHAP and autologous stem cell transplant. Complete remission.
2016	Colon carcinoma, pT3N0M0	Left hemicolectomy
2019/03	Esophageal cancer, cT2N1M0	Neoadjuvant chemoradiotherapy (carboplatin/paclitaxel)
**Medication**		
Lorazepam 1 mg once a day, at night		
Rosuvastatine 5 mg once a day		
Carbasalate calcium 100 mg powder once a day		
Perindopril 2 mg once a day		
**Allergies**		
None		
**Intoxications**		
Smoking	Yes, quit in 2001	
Alcohol	Yes, 6 glasses of alcohol per week	
Drugs	Never	
**Social Context**		
Marital status	Married	
Employment	Manager wholesale glass company: retired	

BMI: Body Mass Index; PTCA: percutaneous transluminal coronary angioplasty; CHOP: combination chemotherapy used for treatment of non-Hodgkin Lymphoma; DHAP/VIM/DHAP: combination chemotherapy used for treatment of non-Hodgkin Lymphoma.

On postoperative day (POD) 2 the patient developed SoB and CP. This was initially deemed reflective of recent post-operative status, however a progression in symptoms warranted additional imagining. A CT on POD 6 showed bilateral PE (left being segmental) with infarction of the right lower lobe and bilateral consolidations suggestive of pneumonia (Fig. 2). Therapeutic fraxiparine 2dd1 7600IE and antibiotics were immediately administered.

**Fig. 1:**
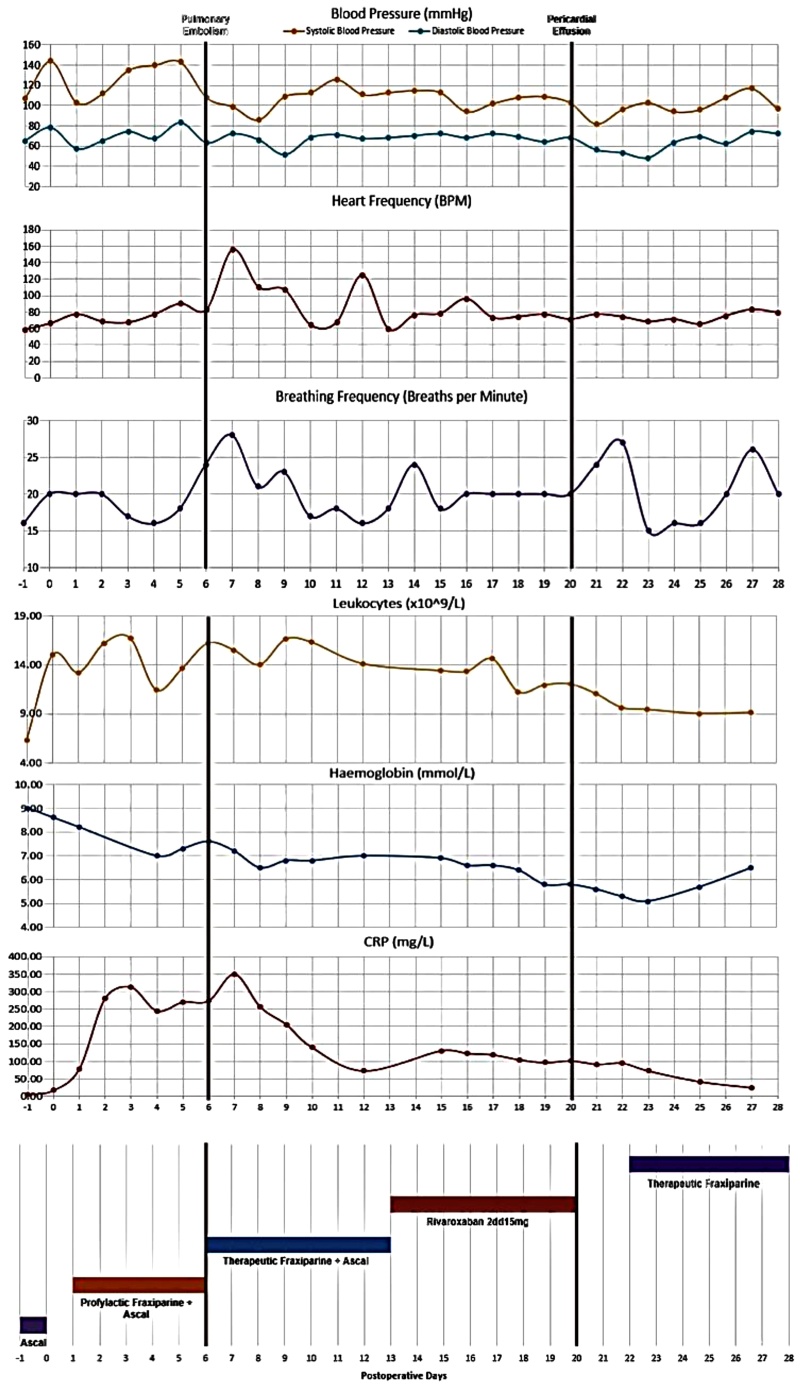
Vital signs, laboratory findings and anticoagulation scheme during postoperative admission; CRP: C-Reactive Protein. Addendum 1: Clinical course.

On POD7 the patient became acutely dyspneic requiring admission to the intensive care with acute respiratory failure secondary to PE and pneumonia. ECG showed atrial flutter. In the blood, a doubling of CRP (350 mg/L) with leukocytes of 15.50 × 10^9^/L and stable hemoglobin were observed (Fig. 1). Optiflow and amiodarone were started, after which respiratory status improved and the arrythmia converted to sinus rhythm. The patient was transferred back to the surgical ward where he continued to recuperate.

On POD13 the patient was switched to Rivaroxaban 2dd15 mg (Fig. 1), as this was deemed a better treatment for the arrhythmia and PE. Due to anxiety quetiapine 1dd12.5 mg, and lorazepam 1dd10 mg were also initiated.

**Fig. 2:**
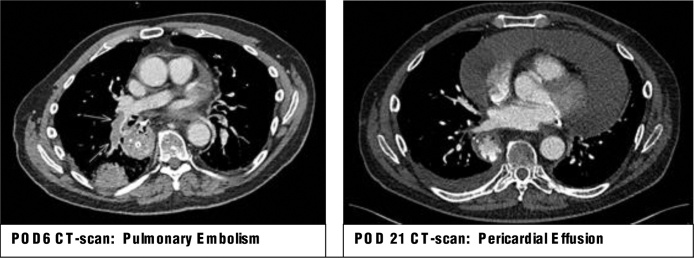
Computed Tomography postoperative Day 6 + Day 21.

Despite treatment, symptoms of SoB and CP continued to progress. Elevated CRP of 100 mg/L and leukocytes of 13.0 × 10^9^/L suggested the symptoms were due to the PE and pneumonia. On POD17 a hypotensive episode (94/68 mmHg) with a regular heart rate of 96bpm was observed. X-thorax showed bilateral pleural fluid and an enlarged heart. On CT a massive PcE was seen with pleural fluid, bilateral consolidations, and mediastinal air pockets suggestive of an anastomotic leakage (AL) (Fig. 2). A transthoracic echocardiogram showed substantial pericardial fluid with no signs of imminent tamponade. Rivaroxaban was stopped, and via pericardiocentesis 1150 mL of fluid was aspirated from the pericardium. Pathological assessment of the fluid showed blood without malignant cells. A cytogram showed erythrocytes, a few leucocytes, no bacteria nor other microorganisms. Therapeutic fraxiparine was resumed 8 h post-pericardiocentesis. Intravenous antibiotics were administered in treatment of the lung consolidations. A small defect at the anastomosis was gastroscopically confirmed, and treated conservatively with antibiotics and nihil by mouth.

Following pericardiocentesis the CP and SoB symptoms improved. X-thorax showed reduced heart contours and pleural fluid. ECG showed sinus rhythm. Laboratory workup showed normalization of infection parameters. The patient was observed for another 5 days without complications before he was discharged from hospital.

## 3 Discussion and conclusion

This is the first reported case of the simultaneous presentation of PE and PcE following esophagectomy. The case poses diagnostic and therapeutic challenges arising from complex anticoagulation considerations.

There are few reported cases of PcE following an esophagectomy [3,4]. This procedure requires the surgeon to work in close proximity to the pericardium in order to dissect and prepare the esophageal tissue for resection. Surgical trauma may precipitate the development of a PcE shortly following surgery. In our case, the PcE was diagnosed on POD20, suggesting that surgical trauma played no direct role in its development.

A systematic review by Pabba et al. [5] presents 7 cases of concurrent PE and PcE in cancer patients, with PcE deemed secondary to malignancy based on cytological fluid analyses. In our case, cytological and pathology results showed no malignant cells. Another common cause of PcE is infection. We found no evidence of elevated white blood cells or microorganisms in cytology, suggesting an alternative cause of PcE.

Our patient received preoperative chemoradiotherapy. Several observational studies on the incidence of PcE following chemoradiotherapy for EC report an incidence of 27.7–57.0% with a median onset of 5.3–12 months following the last chemoradiotherapy session [6,7]. In our case, the PcE was diagnosed approximately 15 weeks after the last chemoradiotherapy session, suggesting that the chemoradiotherapy may have played a role in the development of PcE.

Considering the timeline, SoB and CP symptoms worsened one day after the start of Rivaroxaban. At this time, we see a downward trend in hemoglobin levels - possibly indicating an active bleed. The fact that the patient did not develop a cardiac tamponade despite having almost 1200 mL of blood in the pericardium, suggests that the blood accumulated slowly. Together this draws suspicion towards Rivaroxaban as the PcE-cause. A case series by Cinelli et al. (2019) [8] presents three cases of direct oral anticoagulant (DOAC)-induced PcE in oncologic patients. All three cases showed malignant cells in fluid cytology, suggesting that this may have played a causative role rather than Rivaroxaban.

The current standard of treatment for PE is therapeutic low molecular weight heparin (LMWH). Studies within the general population presented DOACs as an appropriate, patient friendly alternative to LMWH for the treatment of Venous Thromboembolism (VTE). Although encouraging, these studies underrepresented oncologic patients [9]. Two recent randomized controlled trials looked at the effectiveness of DOACs compared to LMWH for the treatment of VTE in cancer patients [10,11]. They showed a decreased incidence of recurrent VTE, with an increased risk of bleeding amongst the DOAC group. This risk was primarily observed in upper gastrointestinal malignancies, with emphasis on esophageal and gastric cancer. Within the esophageal group, bleeding was only noted in unresected esophageal tumors. This suggests that the bleeding in this patient group mainly involved tumour tissue. However, due to the limited number of EC patients in these studies, further research is necessary before firm conclusions can be formulated. Special attention should be given to the uptake of DOACs in EC patients. Studies looking at the effects of bariatric surgery and gastrointestinal resections on DOAC uptake have shown an inadequate absorption of the drug resulting in suboptimal anticoagulation [12]. This is suggestive of a similar problem amongst esophagectomy patients.

Lastly, Rivaroxaban is a substrate of the P-gp efflux transporter, and is metabolized via CYP3A. Medications interactions with these systems can cause changes in medication concentrations [13]. Quetiapine, the antipsychotic which our patient received, acts as a P-gp and CYP3A inhibitor. Both interactions can result in an increased plasma concentration of Rivaroxaban and increasing the risk of bleeding, and potentially playing a role in the development of PcE in our patient.

In conclusion, this case illustrates the diagnostic challenges of SoB and CP in post-esophagectomy patients, as well as the difficulties arising from complex anticoagulant considerations in EC. There is conflicting evidence regarding the safety of Rivaroxaban in EC patients, with no evidence of its uptake following an esophageal resection. In our patient case we suspect that the treatment of PE with Rivaroxaban had a causative role in the development of PcE and based on literature we also suspect that chemoradiotherapy increased susceptibility. Based on current literature and this case study we believe that the use of Rivaroxaban in EC patients should always be conducted in consultation with a coagulation specialist.

## Declaration of Competing Interest

The authors report no declarations of interest.

## Funding

None.

## Ethical approval

Not applicable. Informed consent from patient obtained.

## Consent

Informed consent from patient obtained.

## Registration of research studies

Case study: Not applicable.

## Guarantor

The first and last author (Daniela Jou-Valencia MD^a,1,^*; Frederieke A. Dijkstra MD^a^) accept full responsibility for the study and guarantee it's accuracy.

## Provenance and peer review

Not commissioned, externally peer-reviewed.

## CRediT authorship contribution statement

**Daniela Jou-Valencia:** Conceptualization, Data curation, Writing - original draft. **Frederieke A. Dijkstra:** Supervision, Writing - review & editing.
